# Cheiloscopy in sex estimation: a systematic review

**DOI:** 10.1007/s12024-023-00648-9

**Published:** 2023-05-27

**Authors:** Tânia Chaves, Álvaro Azevedo, Inês Morais Caldas

**Affiliations:** 1 Departamento de Ciências da Saúde Pública e Forenses e Educação Médica, Faculdade de Medicina, Universidade do Porto, Porto, Portugal; 2 Faculdade de Medicina Dentária da Universidade do Porto, Rua Dr. Manuel Pereira da Silva, 4200-393 Porto, Portugal; 3 Epidemiology Research Unit (EPIUnit), Institute of Public Health, University of Porto, Porto, Portugal; 4 Laboratory for Integrative and Translational Research in Population Health (ITR), Porto, Portugal; 5 TOXRUN — Toxicology Research Unit, University Institute of Health Sciences, Gandra, Portugal; 6 Department of Life Sciences, Centre for Functional Ecology (CFE), University of Coimbra, Coimbra, Portugal

**Keywords:** Forensic Science, Criminal investigation, Human identification, Cheiloscopy, Lip prints, Sex

## Abstract

This study aimed to conduct a systematic review to gather evidence to clarify if cheiloscopy can be used in sex estimation and identify the reasons behind the lack of consensus in the scientific community. The systematic review was performed following the PRISMA guidelines. A bibliographic survey was conducted in PubMed, Scopus, and Web of Science databases, restricted to articles published between 2010 and 2020. Studies were selected according to eligibility criteria, and then the study data were collected. The risk of bias in each study was assessed and applied as additional inclusion or exclusion criteria. The results of the articles eligible for analysis were synthesized using a descriptive approach. In the 41 included studies, several methodological flaws and variations between studies that contribute to the discrepancy in results were identified. The data gathered allowed us to conclude that there is no strong scientific evidence to support the use of cheiloscopy in sex estimation, as there is no specific pattern for each sex, which reduces the criminalistic interest of cheiloscopy in estimating this parameter.

**Supplementary Information:**

The online version contains supplementary material available at 10.1007/s12024-023-00648-9.

## Introduction

Although primary methods represent the most reliable human identification methods [1], these techniques cannot always be applied [2], namely due to the lack of such traces at the crime scene. The criminal investigation television series have contributed to this, since the demonstration of crime scene analysis techniques, even if distorted and fanciful of reality [3], alerted criminals to take precautions to avoid traces at the crime scene [4]. Forensic science has evolved to overcome this problem by using different and less-known identification techniques [2], such as cheiloscopy, the name given to the study of lip prints [5].

The lips’ red area mucosa, also called Klein’s zone [6, 7], is covered by several lines, furrows, and lip wrinkles, which vary in number, thickness, length, ramification, and position [8]. These variation combinations give each individual a unique lip pattern [5]. When in contact with a surface, the lips produce a particular mark—the lip print [9]. Lip prints can be found on glasses, paper napkins, certain foods, clothing, photographs, cigarette butts, glass and mirrors, tape, human skin, and open airbags, among others [3, 10–12].

Lip prints’ uniqueness allows for performing a comparative identification between the lip print found at the crime scene and the suspect’s print [13]. However, when this is not possible, lip print analyses may help to estimate other relevant parameters of the individual’s biological profile, namely, sex. Although traditionally applied in cadaver identification [14], sex estimation becomes useful also in the identification of living people, as it can help reduce the number of suspects by half. Many authors have sought to confirm whether sex differences in the lip pattern exist, to determine if cheiloscopy can be used in sex estimation. However, there is no consensus among the scientific community regarding its potential in estimating an individual’s sex [8, 15–19]. The need for more reliable answers from which investigators can draw conclusions and make decisions about the potential of lip prints in estimating sex is important. Although a systematic review on the topic has already been performed [20], this included articles with inadequate statistical analyses and, therefore, biased results, which justifies the need for a new review. Thus, this study aimed to conduct a systematic review to gather evidence and identify possible gaps in the sex estimation cheiloscopy contribution.

## Material and methods

This systematic review was performed according to the Preferred Reporting Items for Systematic Reviews and Meta-Analysis (PRISMA) guidelines [21], and the protocol was registered in PROSPERO with the registration number CRD42022232548.

According to the PICO framework, the following research question was defined: “Can cheiloscopy (intervention) be applied to estimate the sex (outcome) of an individual (population)?”

The bibliographic search was performed in the PubMed, Scopus, and Web of Science Core Collection databases. The search query “((cheiloscopy OR “lip prints”) AND forensic),” present in the title, abstract, or keywords, was used. The publication date was restricted to the last 10 years from the day the search was conducted, i.e., between October 23, 2010, and October 23, 2020, in English, Portuguese, and Spanish languages. The stipulated publication period covers a considerably larger number of studies published in this area compared to the period before 2010, and, therefore, it was not justified to have included studies before the established date. Table 1 shows the search strategy applied in each database. All references obtained were entered and organized in the EndNote X9.3.3 reference management software.

**Table 1:** Search strategy applied in each database

**PubMed**
((“cheiloscopy”[Title/Abstract] OR “lip prints”[Title/Abstract]) AND “forensic”[Title/Abstract]) AND ((2010/10/23:2020/10/23[pdat]) AND (english[Filter] OR portuguese[Filter] OR spanish[Filter]))
Scopus
TITLE-ABS-KEY ((cheiloscopy OR “lip prints”) AND forensic) AND PUBYEAR > 2009 AND (LIMIT-TO (LANGUAGE, “English”) OR LIMIT-TO (LANGUAGE, “Portuguese”) OR LIMIT-TO (LANGUAGE, “Spanish”))
Web of Science Core Collection
(cheiloscopy OR “lip prints”) AND forensic (topic) Refined by: languages: English Timespan: 2010–10-23 to 2020–10-23 (publication date)

After the bibliographic search was performed, duplicate references were removed. In the next step, the studies were assessed for eligibility according to the predefined criteria (Table 2*).* All review studies detected during the bibliographic search or in the reference management software were removed. Then, the remaining studies were assessed for eligibility by reading the title, abstract, and full text. If the full-text article was not available online, the authors were contacted, by email, to provide it. The selection of studies was carried out independently by the three authors. Each one classified each article as “eligible” or “not eligible” at each of the three selection stages. Whenever there was disagreement between the reviewers regarding the eligibility of a study, it was enough for one reviewer to consider the study eligible to move on to the next stage.

**Table 2:** Inclusion and exclusion criteria

**Inclusion criteria**
a) Studies that assess differences in lip pattern between sexes b) Studies estimating sex based on lip pattern
**Exclusion criteria**
a) Studies that do not assess differences in lip pattern between sexes or that do not study the potential of cheiloscopy in sex estimation b) Studies that do not directly relate lip prints to sex c) Reviews d) Letters to the editor e) Abstract not available f) Full text not available

After the selection process, the following data were extracted from the included studies: authors and year, aims, sample size (number of male and female participants), age group, population, lip prints collection method, analysis instrument, classification used, lip area analyzed, statistical analysis method, and results. Data were extracted by a reviewer and confirmed by a second element. Any disagreement was resolved by consensus.

To assess the risk of bias, a list of criteria was developed based on the Critical Appraisal Checklist for Analytical Cross-Sectional Studies from the Joanna Briggs Institute [22]. The list was composed of 10 different domains (Table 3). For each domain, a maximum of five answer possibilities were applied: “Yes,” “Not totally,” “No,” Not reported,” and “Not applicable.” The risk of bias being low/null, medium, high, and uncertain was assigned to those domains answered with “Yes,” “Not totally,” “No,” and “Not reported,” respectively. The three authors were involved in this process. The risk of bias was applied as an additional inclusion or exclusion criterion if it related to the “Statistical analysis” and “Results presentation” domains because those have great relevance to the internal validity of the study. So, an article presenting a high risk of bias in at least one of these two domains was excluded.

**Table 3:** Risk of bias assessment criteria

1. Is the aim well defined?
2. Are the characteristics of the study population clearly specified?
3. Are the inclusion or exclusion criteria of participants specified?
4. Is the methodology presented and appropriate?
5. Was intra-rater reliability assessed?
6. Was inter-rater reliability assessed?
7. Was the statistical analysis applied adequate and well explained?
8. Is there an explicit and error-free results presentation?
9. Does it answer the study aim?
10. Are the conclusions based on the study results?

The results of the individual studies were synthesized using a descriptive approach. A quantitative synthesis, such as meta-analysis, was not performed due to the heterogeneity of lip print collection techniques, lip areas analyzed, classification systems, and statistical methods applied among the studies, which makes them not comparable with each other. The results were summarized in a table, in which the remaining variables of interest from each study were also presented. The articles were arranged in the table in descending order of low risk of bias. In addition, a graph representing the different methods of collecting and analyzing lip prints, the classifications used, and the different areas of the lip analyzed by the studies was presented.

## Results

### Study selection

**Fig. 1:**
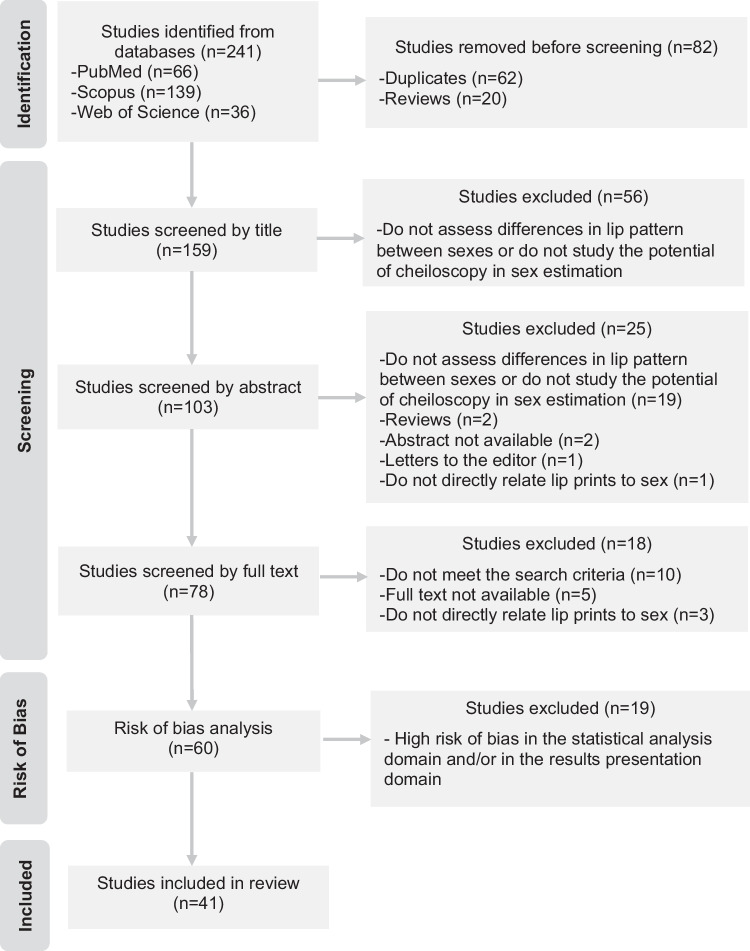
Flow diagram representing the article selection process

As recommended by the PRISMA guidelines, the selection of studies was documented in detail in a flow diagram (Fig. 1). The search strategy identified 241 studies. After removing duplicates, 119 articles were excluded by applying the eligibility criteria. Given the statistical analysis risk of bias attributed and results presentation domains, additional 19 articles were excluded. Thus, 41 studies were included in this systematic review.

### Collection and analysis of lip prints

The procedure employed during the collection and analysis of lip prints varied significantly between studies. Regarding the method applied to collect the participants’ lip prints, it was possible to identify seven different collection methods (Table 4).

**Table 4:** Lip print collection methods applied by the different studies

Method 1	*• Lipstick* application on the lips *•* Application of *cellophane tape* on the lips in order to register the lip print *•* The cellophane tape is pasted on *paper*
Method 2	*• Lipstick* application on the lips *•* Participants are asked *to rub* their lips in order to spread the lipstick evenly *•* The lip print is recorded directly on *paper*
Method 3	*• Lipstick* application on the lips *•* Participants are asked *to rub* their lips in order to spread the lipstick evenly *•* Application of *cellophane tape* on the lips in order to register the lip print *•* The cellophane tape is pasted on *paper*
Method 4	*• Lipstick* application on the lips *•* The lip print is recorded directly on *paper*
Method 5	*• Lipstick* application on the lips *•* Participants are asked *to rub* their lips in order to spread the lipstick evenly *•* Lip print is recorded directly on a *transparent support*
Photographs	*•* Photographs are taken directly to the participants’ lips
Print development	*•* The participant is asked to mark their lip print on a surface and then it is developed using developers

In lip print analysis, two types of instruments were used: a magnifying lens, including the magnifying glass or the stereomicroscope (hereinafter referred to as the direct method), and image editing software, such as Adobe Photoshop, for example (hereafter referred to as the indirect method).

To analyze and classify the labial grooves, several authors have chosen the Suzuki and Tsuchihashi (S&T) classification [23]. However, in several studies, this classification was used with alterations, including the addition of Type I' to Type I, the omission of Type I' and V, and the addition of other pattern types. Some authors developed their classification.

**Fig. 2:**
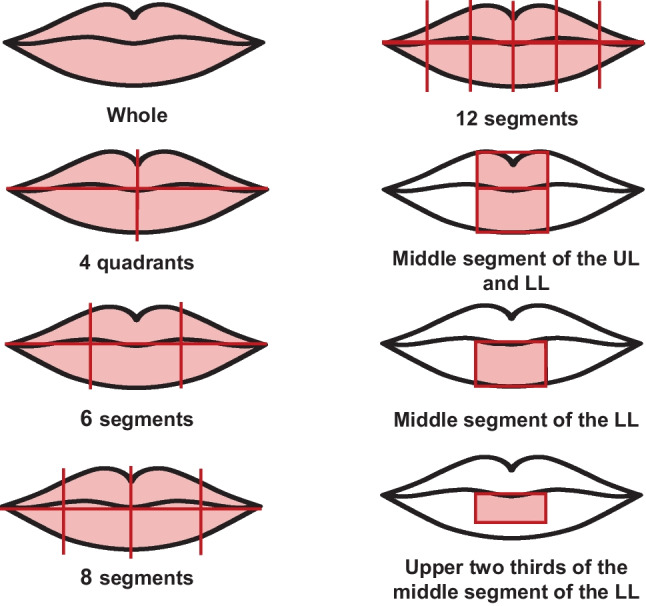
Illustration of the different lip zones analyzed by the studies. (UL, upper lip; LL, lower lip)

Greater heterogeneity was observed in the lip area chosen for analysis. The choices included analyzing the whole lip, without segmental division, or dividing into four, six, eight, or twelve segments, most often, numbered clockwise. The area to be analyzed was also limited to more restricted areas (Fig. 2).

### Characteristics of eligible studies

The variables of interest in the included studies are described in Supplementary Table 1.

The sample size in the different studies ranged from 20 to 1399 participants, from all age groups and from different geographical and ethnic backgrounds, covering the five continents. The Indian population was the most studied, represented by more than 65% of the articles (27/41).

**Fig. 3:**
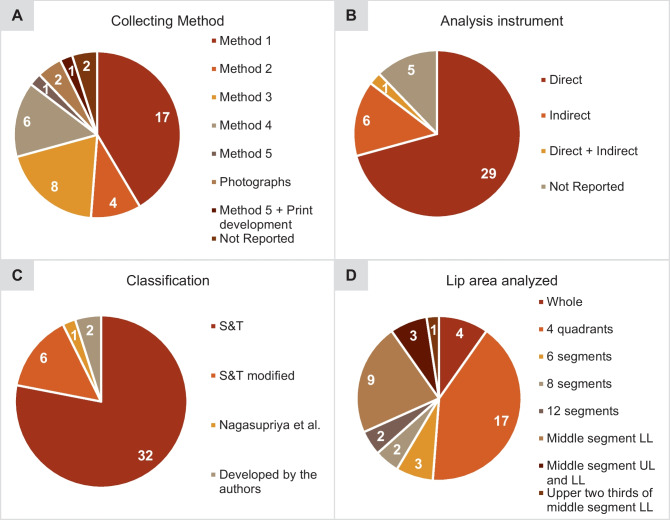
Distribution of lip print collection and analysis techniques among the studies: **A** lip print collection method; **B** analysis instrument; **C** classification; **D** lip area analyzed. (UL, upper lip; LL, lower lip)

In the lip print collection, method 1 was the most applied, having been employed in 41% of the studies (17/41). The direct method of lip print analysis was used in about 70% of the studies (29/41). More than half of the studies (32/41) adopted the S&T system to classify the labial grooves, followed by the same modified classification (6/41). In two investigations, the authors developed their classification. In one study, the classification developed by the authors of another study was used. The sex estimation classification proposed by Vahanwala et al. [24] was also applied in five research studies. Regarding the area of the lip considered for analysis, in about 68% of the studies (28/41), the whole lip was analyzed, mostly divided into quadrants (17/28), and in about 32% (13/41), a more restricted part of the lip was studied (Fig. 3).

### Risk of bias

**Fig. 4:**
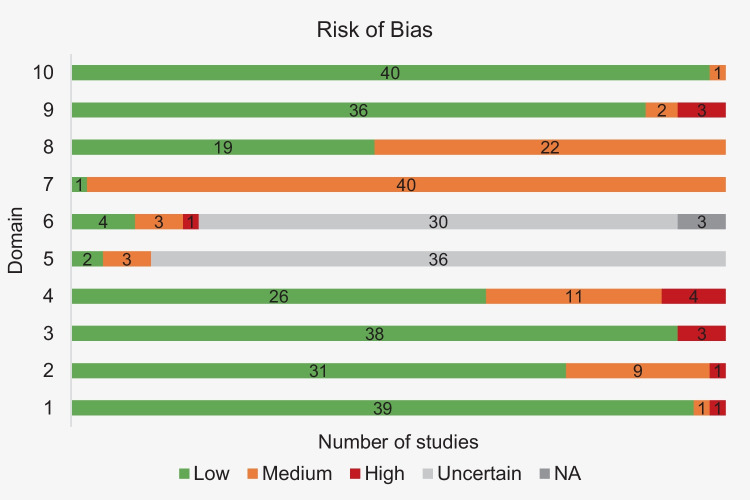
Frequency of studies by level of risk of bias and for each domain

The risk of bias in the included studies is presented in detail in supplementary Table 2. Figure 4 summarizes the information in supplementary Table 2 with the frequency of publications by the risk of bias and for each domain.

In domain 1, almost all studies achieved a low risk of bias, and only one article was classified as high risk [25]. Regarding the characterization of the population, 10 studies failed to present the necessary data, and, within these, one article did not specify the distribution of participants by sex [26], leading to a high risk of bias. Still, most of the articles (31/41) specify all the population statistics. The same was observed in domain 3, with 38 studies presenting the inclusion or exclusion criteria of the individuals. In the remaining three publications, the criteria applied were not reported [12, 27, 28] and were therefore classified as high risk. In domain 4, referring to the methodology presentation, 26 studies present all the steps and a valid classification (low risk of bias). Eleven studies received a medium risk of bias, mostly for using the modified S&T classification. Four articles were classified as high-risk, one of them due to the lack of description of the methodology employed [29] and the others for the use of a classification developed by the authors themselves or by others [26, 27, 30]. Regarding domain 5, intra-rater reliability was assessed in five studies. In two of them, a valid method was used, and the test value was presented (low risk of bias). In the other three, the method applied and/or the test value is not presented (medium risk) [18, 31, 32]. In domain 6, inter-rater reliability was calculated in eight studies, but only four received a low risk of bias. Three studies achieved a medium risk of bias, and one study was classified with high risk due to the application of an invalid method in calculating reliability [33]. Regarding the statistical analysis, a large percentage of studies (40/41) did not pass beyond the medium risk of bias. Only one study received low risk [8], using an appropriate inferential analysis that was well explained and that met the necessary assumptions. In the presentation of the results, more than half of the studies (22/41) failed on some relevant points, which is acceptable for a medium level of risk of bias. A low risk of bias was assigned to 19 studies because they presented the results explicitly and with the required values. In domain 9, only three articles did not answer the proposed objectives, which conditioned the classification as high risk [7, 26, 29]. Still, most of the studies achieved a good rating in this domain. The same was true in domain 10, where 40 articles achieved a low risk of bias. Only one study did not base all its conclusions on the results obtained [7].

**Fig. 5:**
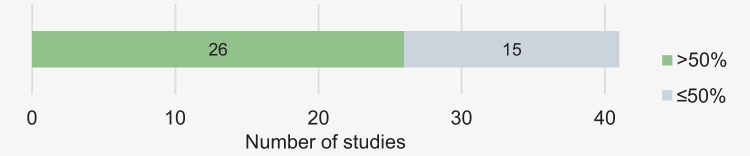
Frequency of studies with more and less than 50% of domains with low/null risk

Overall, most studies, i.e., 26 studies, achieved low/null risk of bias in more than half of the domains (Fig. 5).

### Results of the individual studies

Thirty-two studies out of 41 proved that lip pattern is different between sexes, while nine showed just the opposite (supplementary Table 1).

In the group of the first four articles, the highest ranked in terms of risk of bias, differences in lip patterns between sexes were found in three studies, and one study [8] showed no association between lip pattern and sex of the individual (Fisher’s exact test, *p* = 0.54). In the study by Randhawa et al., the sample was divided into three age groups, and although the authors found differences between sexes in all three groups, these differences were more significant in the age group between 21 and 40 years old. Similarly, the percentage of correctly identified males and females, according to the sex estimation classification proposed by Vahanwala et al. was higher in this age group, achieving an accuracy of 76% [34]. These results are in line with the results of Ramakrishnan et al. ($\chi$^2^ = 99.826; *p* < 0.001) who, based on the same classification, also correctly identified the sex of a large number of individuals belonging to a similar age group [17]. In the same way of reasoning, Mantilla Hernández et al., who analyzed individuals between the ages of 18 and 25 years, also identified differences in the patterns of each sex [35]. Herrera et al., on the other hand, who, like the previous authors, analyzed the middle segment of the lower lip, found no evidence of an association between lip print and sex (Fisher’s exact test, *p* = 0.54) in a sample of participants aged 18 to 71 years [8].

In the plateau below, of low risk of bias, are nine studies, and in all of them, sex differences were proven to exist. Here, the authors included participants between the ages of 18 and 40, except for Moshfeghi et al. [36] and Dey et al. [37] who, in addition to this age range, analyzed individuals at earlier and later ages. In the seven studies that included participants aged 18 to 40 years, sex differences were identified across the entire lip area considered for analysis, except for the work by Basheer et al. where, although the four quadrants of the lip were analyzed, sex differences were only found in the upper lip [38]. In the study by Moshfeghi et al., whose participant’s ages ranged from 13 to 70 years, statistical differences were found only in the right segment of the lower lip ($\chi$^2^, *p* = 0.018) [36]. Similarly, in the study by Dey et al., where the sample had individuals above 15 years of age, differences were only found in certain segments of the lower lip. In the Oraon tribals, the differences were observed only in the fourth quadrant ($\chi$^2^ = 14.39; *p* < 0.05), and in the Bengalee Hindus, the differences were observed in the third ($\chi$^2^ = 24.07; *p* < 0.05) and fourth quadrants ($\chi$^2^ = 27.65; *p* < 0.01) [37].

In the following set of 13 studies, eight identified differences in lip patterns between the sexes and five showed the opposite. In the study by Oliveira et al., the authors found statistical differences between sexes with chi-square test (*p* < 0.001) when analyzing the entire lip, but an analysis by the eight segments revealed that differences only existed in regions of the lower lip, more specifically, in segments 6 ($\chi$^2^, *p* = 0.016) and 8 (Fisher’s exact test, *p* = 0.008) [39]. The same was observed in the population of Egypt in the study by Abdel Aziz et al., where, at the quadrant level, the statistical differences were found in quadrants 3 ($\chi$^2^ = 12.616; *p* = 0.008) and 4 ($\chi$^2^ = 14.156; *p* = 0.005). In the Malaysian population, no differences between sexes were observed when analyzing the lip as a whole ($\chi$^2^ = 7.507; *p* = 0.186), but analysis by quadrants identified differences in the second quadrant ($\chi$^2^ = 17.498; *p* = 0.001), belonging to the upper lip [40]. The results of Bharat Kumar suggest, applying descriptive analysis, the presence of different patterns between sexes in quadrants 2, 3, and 4 [32]. Regarding the proportion of each lip pattern in each sex, some authors have found differences in specific pattern types. In the Brahmins community, for example, studied by Vats et al., differences between sexes were detected in the frequency of type I', II, III, and IV of the S&T classification (*Z*-test, *p* < 0.05). In the Jats community, these differences were in patterns I', II, III, IV, and Y (*Z*-test, *p* < 0.05), the latter a type added by the authors to the S&T classification, which represents the mixture of two or more patterns. In the scheduled castes community, it was types I, I', II, III, and V that varied between sexes (*Z*-test, *p* < 0.05). In the set of three Indian communities, all patterns showed different proportions in each sex [41]. Manikya et al. found statistical differences in the proportion of type III in the Karnataka ($\chi$^2^, *p* = 0.03) and Kerala population ($\chi$^2^, *p* = 0.004), and type I in the Manipur population ($\chi$^2^, *p* = 0.02) [42]. In the investigation by Yendriwati et al., statistical sex differences were observed in type IV ($\chi$^2^, *p* = 0.007) [43]. When separating by age groups, Multani et al. found significant differences between sexes in individuals aged 26–35 years ($\chi$^2^ = 8.32; *p* < 0.001) and in individuals over 45 years $\chi$^2^ = 7.84; *p* < 0.001), and very highly significant differences in the age group 15–25 years ($\chi$^2^ = 11.64; *p* < 0.0001) and 36–45 years ($\chi$^2^ = 10.43; *p* < 0.0001). The authors also used the classification of Vahanwala et al. to assess the accuracy of cheiloscopy in sex estimation, which was highest in the 36–45 age group (90.5%). The lowest accuracy was obtained in the oldest individuals, aged over 45 years. Nevertheless, in any of the age groups, the accuracy of cheiloscopy in sex estimation was equal to or greater than 80% [44]. In the study by Manikya et al., 61% of males and 59% of females were correctly identified based on the same-sex estimation classification, which represents an accuracy of 60%, for the age group studied, i.e., 18 to 23 years [42].

In the following group of articles, six studies proved that there are differences between sexes, and three showed that it is not possible to distinguish sexes based on lip prints. Among the studies that confirmed the potential of lip prints in sex estimation, the age of the participants was similar, ranging from 15 to 35 years old. Differences were identified across the entire lip area considered for analysis, except in the study by Priya et al., where differences were detected by chi-square test only in the right segment of the middle part of the upper lip (RU1) (*p* = 0.001) and the left segment of the middle part of the lower lip (LL1) (*p* = 0.04) [45]. In the studies that showed no differences between sexes, it is possible to see, except for the study by Yandava et al. [7], greater heterogeneity in the participants’ ages, as individuals between the ages of 3 and 70 years old were included.

In the penultimate group, three articles are included, whose results indicate the presence of sex differences. In the study by Negi and Negi [27], in particular, the relation found by the authors between sex and lip pattern was that males were more likely to have type II ($\chi$^2^ = 13.480; *p* = 0.001). The same came to be demonstrated in the other two articles [28, 46], as type II was the dominant pattern in males. Likewise, type I was the dominant pattern in females in all three studies, although in the first study, type I represents complete and incomplete vertical lines. Although the participants’ ages are not reported in the study by Negi and Negi [27], nor is the population in the study by Bai et al. [46], it is possible to state that the first and second studies in this group had individuals from the same country and achieved similar results, and the second and third studies in the group had individuals with the same ages, even though they might be represented by different proportions and also achieved the same result.

In the last set, there is again consistency among the studies regarding the existence of sex differences. At the pattern level, Padmavathi et al. found differences between sexes ($\chi$^2^, *p* < 0.05) only in the patterns that the authors classified as “dots” (D), “reticular” (R), and “complex pattern” (CP). Moreover, this difference was observed only in the upper lip, leading them to conclude that only the upper lip can help in estimating the sex of an individual [26].

## Discussion

Most studies indicate that there are differences in lip patterns between males and females. However, nine studies demonstrated the opposite. After a detailed analysis, it was found that there is no specific factor that explains why nine studies found no differences between sexes and the others found differences between sexes, apart from methodological flaws related to the sampling method, which may have conditioned the result achieved by each study. At the same time, several factors may explain the divergence of results within the group of studies that found sex differences and within the group of studies that found no differences.

One of the factors that may have influenced the result of each study concerns the difficulties in analysis arising from the age heterogeneity of the samples. In several studies, the authors included participants from a wide age range [8, 17, 33, 36, 41, 47–49], disregarding age stratification. This procedure may induce errors in the print analysis, because depending on the individual’s age and, therefore, the developmental stage of the lips, the labial lines may show some blurring, making it difficult to correctly analyze or differentiate the sex of the individual. According to Mamandras, between 8 and 18 years old, the lips vary constantly in length and thickness, reaching their maturity in late adolescence. In females, the upper lip reaches maturity at 14 years old and the lower lip at 16 years old, whereas in males, both the lower and upper lips reach the end of development at 18 years old [50]. Around 30 years old, the signs of aging begin to appear around the mouth, but the lips maintain their tone [51]. From 40 years old and with advancing age, wrinkles grow in the skin adjacent to the lips, the intercomissural distance increases, and lip height decreases. These age-related effects do not change the type of lip furrows [52], but the change in the natural lip anatomy can make it difficult to identify the lip pattern and, subsequently, lead to errors in registration. Therefore, it is possible that up to 18 years old and from 40 years old onwards, there is difficulty in analyzing lip prints, caused by the growth and aging of the lips, respectively. It seems that the 18 to 40 age group is the most effective in analyzing and identifying the individual’s lip pattern. The study by Randhawa et al. precisely proved this, since in the sample distributed over three age groups, the greatest accuracy of cheiloscopy in sex estimation was obtained in the group of individuals aged between 21 and 40 years old [34]. Similarly, in the study by Multani et al., the highest percentage of correct identifications was achieved in the age group of 36 to 45 years old. In individuals above 45 years old, there was greater difficulty in correctly identifying the sex [44]. Chaudhari et al. mentioned lip changes due to age as the possible reason for the results obtained [49].

There is a notorious tendency of researchers to justify the inexistence of sex differences with the age heterogeneity of the sample. However, from the results of the present review, this reason may not be enough to explain the results since several studies included individuals outside the age range of 18 to 40 years old and were able to find differences between sexes. Therefore, it is intended to clarify that difficulties in analysis due to lip growth and aging, and subsequent error in recording, occur regardless of whether there may be differences between sexes. However, to avoid their influence on the results, it would be more appropriate for the studies to be carried out in age-stratified samples.

On the other hand, it is possible to see, in several studies, marked sample differences between the proportion of male and female participants concerning the target population [7, 36, 53–56], which may again influence the results. In these cases, the sample size of one of the sexes may have been insufficient and may have conditioned obtaining a different predominant pattern than would have been identified if the sample had been representative.

Another factor that may have influenced the results is the use of a sample consisting of several population groups [29, 45, 53]. In the study by Peeran et al., for example, the inability to differentiate the various ancestral patterns among the sampled population is even presented by the authors as the main limitation. The southern Libyan population, the region where the study was conducted, is composed of individuals of various ancestries [53], which may have been a decisive factor in the lip print analysis since there are studies that showed that the lip pattern varies between different population groups [12, 42]. Thus, if this hypothesis is proven, it would be more reliable for the analysis of sex differences to be performed among people of the same ancestry or population group.

Thus, the difficulty in the analysis caused by age heterogeneity, the lack of sample representativeness, and the diversity of ancestry or population groups may have influenced the results of different studies.

Considering the group of studies that found differences between sexes and the group of studies that did not find the same results, it is possible to verify that within each group, and even between groups, the results differed, especially in the predominant lip pattern in each sex. These discrepancies can be explained by several factors, especially by the geographic origin of the sample. As mentioned earlier, there seems to be an evidence that lip pattern varies between individuals from different population groups. Thus, if this is proven, it is to be expected that the predominant pattern in males and females may vary depending on the population group to which they belong.

The sampling methodology also explains the different results obtained, starting with the sample size, which ranged from 20 to 1399 participants, and only one study selected participants randomly [17]. It is natural that in a sample of 20 individuals, the same predominant patterns may not be found as in a sample of a thousand individuals, for example, especially when dealing with convenience samples, where people are not randomly selected. In fact, given the variability of the factors under study, selecting the appropriate sample size is an important step to make an inference of an outcome to the population. The sample size should be calculated in advance, considering the population size and the frequency of events, to ensure that the results obtained from the sample can be generalized to the population [57]. Understandably, it is limiting to conduct studies with representative samples of populations as large as those studied by the included articles. However, it is important to consider the detriment of conducting studies of reduced power or the risk of obtaining biased results.

**Fig. 6:**
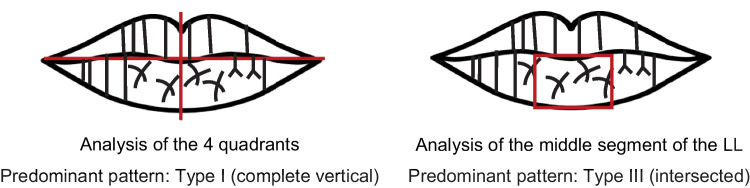
Representative illustration of the influence of the lip area under study in determining the predominant pattern. (LL, lower lip)

Another factor that can explain the differences in the predominant pattern of each sex among the various studies presented is the area of the lip considered to classify the labial grooves, which varied between the studies. With such diverse analysis areas and knowing that the type of lines and their frequency vary throughout the labial mucosa [58], it is possible that such a factor explains the discrepancies observed between the studies. So, while some investigators studied the numerical superiority of the type of lines present on the whole lip, segmented or not, others only studied the numerical superiority of the type of lines found on a restricted lip portion. Thus, it is to be expected that when analyzing the whole lip print, one will find a particular type of pattern that may not be the same as that found when analyzing only a smaller portion (Fig. 6).

**Fig. 7:**
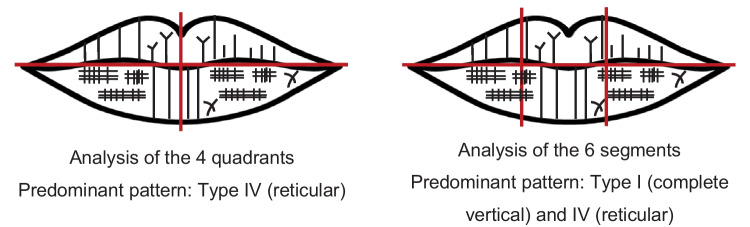
Representative illustration of the influence of the segmentation of the lip area under study in determining the predominant pattern

The same is true when the whole lip is analyzed and segmented in different ways. That is, analyzing the lip divided into four parts, for example, produces different results from those achieved when analyzing the lip divided into six, as exemplified in Fig. 7.

**Fig. 8:**
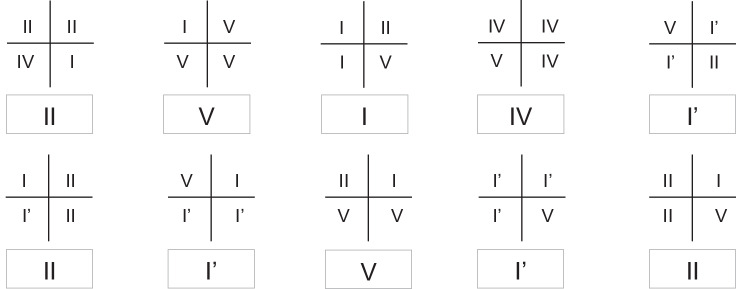
Representative illustration of lip print analysis by quadrants. Within each rectangle is shown the predominant pattern of the lip print represented above

Another parameter that can make a difference is that some authors determine the predominant pattern according to the pattern that prevailed in the total segments of each sex, while others determine the predominant pattern of each sex according to the pattern that prevailed. For example, in the study by Ramakrishnan et al., a sample of 100 individuals was analyzed. Lip prints were divided into four quadrants, and in the results, the authors report that 33% of the total quadrants of the males showed type I [17]. This calculation, for the total number of quadrants, gives a different result from that which would be found if the determination of the lip pattern were made considering the predominant pattern of each of the individuals of the sex under analysis. Figure 8 provides an example. In a sample of 10 males, the lip prints are divided into four quadrants, and the analysis is done according to the numerical superiority of the type of lines observed in each quadrant. Next, the lip pattern of the print under analysis is determined, considering the pattern that dominated in each of the four quadrants. The conclusion is reached that types I' and II are the predominant patterns in males. However, when considering the totality of the quadrants, the pattern that prevails in a larger number of segments is type V. In 11 of the 40 quadrants, type V dominates. This leads to the conclusion that type V is the predominant pattern in males. As can be seen, depending on how one intends to calculate the predominant pattern, the result may vary.

During the analysis of lip prints, although most authors used the full S&T classification, it was also possible to identify some studies that applied the modified classification [33, 36, 41, 47, 53, 59]. The change in classification, mostly by adding or omitting some types of lines, results in data that would differ from those found if the original classification was applied. This further contributes to the discrepancy in results between studies [25, 40, 41, 53].

Regarding the sex estimation classification proposed by Vahanwala et al. [24], its correct use by the studies raises doubts, since, according to the classification authors, it should be applied to the analysis of the four quadrants of the lip, and not to different or smaller areas, as was done in three studies [34, 42, 44]. Now, using a classification developed to be applied to all four quadrants of the lip, in completely different zones, will produce invalid results. To clarify whether this sex estimation classification could be applied to different areas of the lip, other than the four quadrants, the lead author of the classification was contacted, in October 2021. However, as of the date of submission of this paper, no response has been obtained.

The heterogeneity of the techniques used to collect and analyze lip prints may also have been determinant in obtaining different results, since the way these techniques are performed may affect the correct reading of the prints and, consequently, influence the results of each study [7, 17, 18, 32, 48, 60].

Regarding collection, the method employed is a fundamental step to ensure print quality. Recording the lip print is a technique-sensitive task and, therefore, depending on how the print is collected, its quality may vary. Thus, choosing the most appropriate method is essential to ensure the success of the analysis. Costa and Caldas [61] tested methods 1 to 4 and found that the application of lipstick, without rubbing the lips, followed by transfer to cellophane tape (method 1) is the method that provides the best lip print reading. This was the collection method most used by the articles included in the systematic review. A 2010 study comparing, among other collection techniques, methods 2 and 3, showed that the latter is the most appropriate because of the good quality of the print, low technical difficulty, and speed of the procedure [62]. Regardless of the advantages they may present, the main limitation reported by studies using conventional methods is the amount of lipstick applied that, in excess, can decrease the print quality [54]. Evidence shows that prints taken with a thinner layer of lipstick have better quality [63].

The pressure applied during collection and the direction can also alter the appearance of the prints [58] and consequently affect correct identification. Human lips are naturally mobile [18], and therefore, the pattern of lip wrinkles depends on how the muscle relaxes to produce the print [5]. When the muscle relaxes, the mouth remains closed, and a lip print with well-defined lines is produced. On the contrary, in the open-mouth position, the lines are less perceptible and therefore more difficult to classify [64]. The lips’ mobility explains why the same person can produce lip prints with different appearances according to the pressure and direction applied. To overcome this limitation and to avoid errors in classification, some researchers have used the photography technique to record lip prints instead of the traditional lipstick and paper recording method [18, 31]. From a practical point of view, photography has a great advantage in that it is more convenient for the subject since he does not need to apply lipstick, as would be the case with conventional methods. Suspects of a crime often resist the collection of their biometric data. Having to apply lipstick to them and take their lip print by conventional methods would be a difficult task, and worse if more than one print must be taken to ensure the best quality. Furthermore, taking the print using cellophane tape may be painful for the individual as it may cause small lesions on the lips [40]. Therefore, several authors suggest photography as the most appropriate method for taking lip prints [31, 65]. To do so, it is very important to create good lighting conditions, as mismatched shadow and light areas may influence the quality of the images [8].

Regarding analysis, the direct method was the most used, essentially because it is very practical and simple. But, if on the one hand, it is easy to use, on the other hand, it may not offer the best visualization of the prints. In latent lip prints, for example, the use of alternate light sources, like blue or green light, may allow for better results [66]. Similarly, other techniques involving filters, polarized filters, contrast, and other imaging software techniques have also offered good print visualization [67]. As for the indirect method, image-editing software allows for improving the visualization of prints by adjusting brightness, color, contrast, or enlarging details [48, 68]. In this way, the same lip print, with some imperceptible or overlapping details, may see its quality improved with the use of image editing software, while the simple use of the magnifying lens would not allow it. Thus, the analysis method applied may also influence the results since better visualization of the prints will certainly lead to an increase in correct analyses. Other imaging software, such as Adobe 7.0, has already been used by other authors, who reported better visualization, ease in identification, and recording of the lip print pattern [69].

Despite the discrepancy of results and most studies indicating the existence of differences between sexes, namely those better ranked as to the risk of bias, it is important to retain that there is no specific pattern for each sex, which makes the effectiveness of reconstructive analysis for sex estimation relative and of questionable practical utility.

## Conclusion

This systematic review identified several methodological flaws and variations between studies that contribute to the discrepancy in results and, therefore, to the lack of consensus regarding the implementation of cheiloscopy in sex estimation.

The data gathered allowed us to conclude that there is no strong scientific evidence to support the use of cheiloscopy in sex estimation, as there is no specific pattern for each sex, which reduces the criminalistic interest of cheiloscopy in estimating this parameter. In any case, if lip prints are employed in the sex estimation, this process should be carried out with caution, as a false estimation can harm a forensic investigation.

## Key points

1. Sex estimation using cheiloscopy has been suggested.
2. Some data suggest sex estimation using cheiloscopy cannot be done.
3. Methodological differences can explain the different results.
4. Our results point to no strong scientific evidence to support this practice.

### Supplementary Information

Below is the link to the electronic supplementary material.

Supplementary file1 (DOCX 234 KB)

Supplementary file1 (DOCX 62 KB)

## Data Availability

Data are available at the supplementary tables.
