# Supplementary material for: Cheiloscopy in sex estimation: a systematic review
Source: Forensic Sci Med Pathol. 2023 May 27;20(1):280–92. doi: 10.1007/s12024-023-00648-9 (PMC10944408; doi:10.1007/s12024-023-00648-9)
Supplement: Supplementary file 1 — Supplementary file1 (DOCX 234 KB) [file 12024_2023_648_MOESM1_ESM.docx]

Supplementary table 1 – Results of Individual Studies on Cheiloscopy

| Authors and year | Sample (M/F) | Age group (years) | Population | Print collection method/Analysis method | Classification/  Lip area analyzed | Method of statistical analysis | Results | |
| --- | --- | --- | --- | --- | --- | --- | --- | --- |
|  |  |  |  |  |  |  | **Are there differences between sexes?** | **Description** |
| Randhawa *et al.*, 2011 (1) | 600 (289/311) | All ages | North India | 3/Direct | S&T and Vahanwala *et al.*/ Upper two thirds of the middle segment of the LL | Chi-square Test | 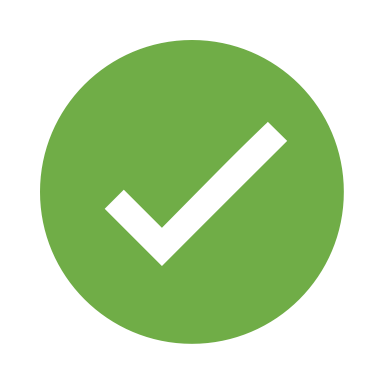 | 41.52% of males: type III; 59.48% of females: type I;  Group 1 (1-20 years): 90.27% of females and 29.48% of males correctly identified; 48 females and 40 males with type I;  Group 2 (21-40 years): 89.4% of females and 65.9% of males correctly identified; 102 females with type I and 71 males with type III;  Group 3 (>41 years): 65% of females and 57.14% of males correctly identified; 35 females with type I and 33 males with type III;  Accuracy of cheiloscopy in sex estimation: 58.67% (group 1), 76% (group 2) and 61.33% (group 3);  Sex differences: sig. in g1 (p=0.001), very highly sig. in g2 (p<0.0001) and highly sig. in g3 (p<0.001) |
| Mantilla Hernández *et al.*, 2015 (2) | 60  (30/30) | 18-25 | Santander, Colombia | 3/Direct | S&T/Middle segment of the LL | Descriptive analysis | 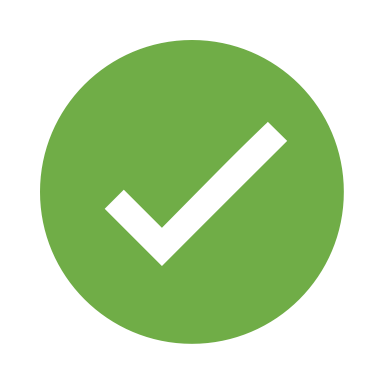 | 40% of males: type I’;  36.6% of females: type V;  Differences were found in the pattern of each sex |
| Ramakrishnan *et al.*, 2015 (3) | 100  (50/50) | 18-50 | Bangalore, India | 5 + print development /Indirect | S&T and  Vahanwala *et al.*/4 quadrants | Chi-square Test | 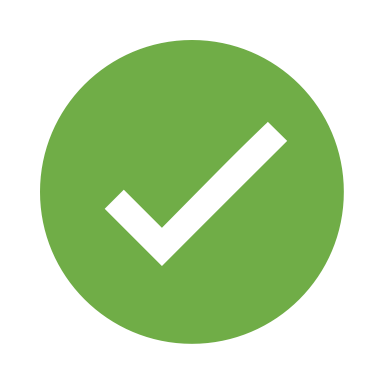 | 33% of the total male quadrants: type I;  61% of the total female quadrants: type I;  Statistically sig. association between lip print and sex (ꭓ^2^=99.826; p<0.001);  Examiner 1 correctly identified the sex of 94 prints; examiner 2 correctly identified the sex of 93 prints |

(Continues)

| Authors and year | Sample (M/F) | Age group (years) | Population | Print collection method/Analysis method | Classification/  Lip area analyzed | Method of statistical analysis | Results | |
| --- | --- | --- | --- | --- | --- | --- | --- | --- |
|  |  |  |  |  |  |  | **Are there differences between sexes?** | **Description** |
| Herrera *et al.*, 2018 (4) | 50  (25/25) | 18-71 | Brazil | 3/Indirect | S&T/Middle segment of the LL | Fisher’s Exact Test | 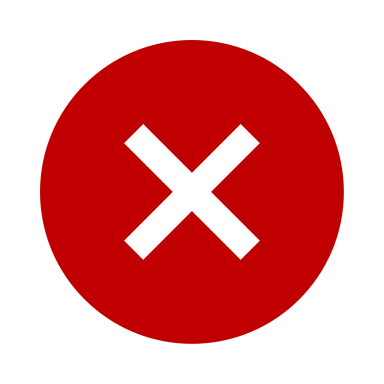 | 56% of males: type III;  32% of females: type III;  32% of females: type I;  There is no evidence of association between types of lip grooves and sex (p=0.54) |
| Badiye and Kapoor, 2016 (5) | 400 (200/200) | 18-25 | Marathi, India | Photographs /Indirect | S&T/Middle segment of the UL and LL | Chi-square Test | 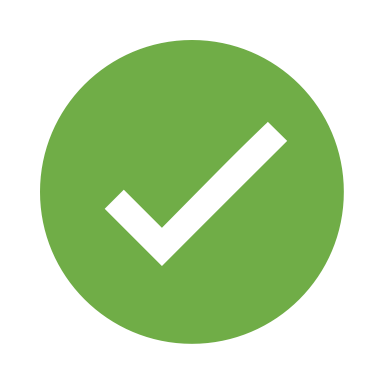 | 32% of the total male segments: type II;  32.5% of the total female segments: type IV;  ꭓ^2^=36.86;  There is a significant association at a level >95% and >99% between sex and lip print types, thus proving the potential of lip prints for recognition of the sex of the donor |
| Moshfeghi *et al.*, 2016 (6) | 96  (22/74) | 13-70 | Tehran, Iran | 1/Direct | S&T modified/6 segments | Chi-square Test | 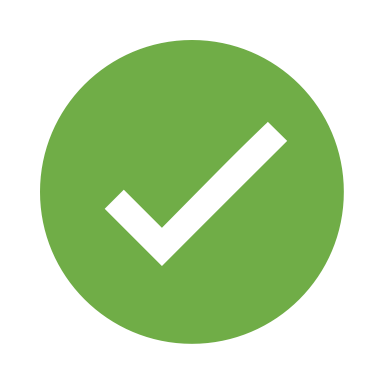 | 39.39% of males: type V;  31.31% of females: type V;  There are no statistically sig. differences between sexes in different areas; there are only in the RL segment (p=0.018) |
| Tarvadi and Goyal, 2016 (7) | 100  (50/50) | 18-25 | Rajasthan, India | 5/Direct | S&T/Middle segment of the UL and LL | Descriptive analysis | 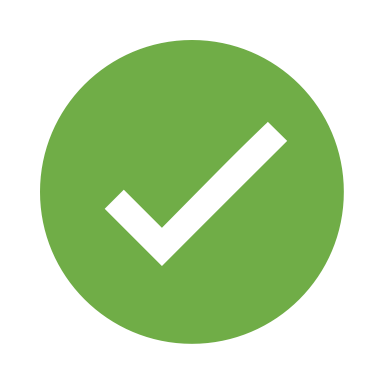 | 19 males with type II;  19 males with type III;  17 females with type III;  The lip pattern distribution was different in both genders |
| Basheer *et al.*, 2017 (8) | 858 (471/387) | 18-30 | North Kerala, India | 1/Direct | S&T/4 quadrants | Descriptive analysis | 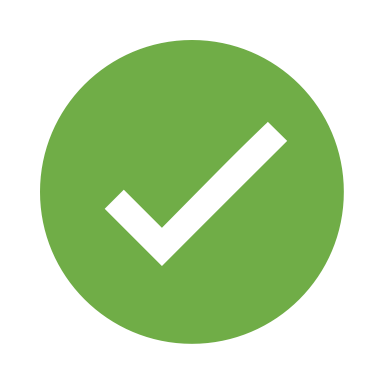 | Predominant pattern in males: type II in UL and type I in LL;  Predominant pattern in females: type IV in UL and type I in LL;  The upper lip can help in sex estimation |
| Kapoor and Badiye, 2017 (9) | 200 (100/100) | 18-25 | Marathi, India | Photographs /Indirect | S&T/4 quadrants | Chi-square Test | 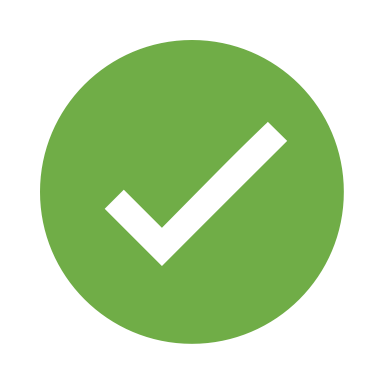 | 29.75% of the total male quadrants: type I;  35.75% of the total female quadrants: type III;  There are statistically sig. differences between sexes in all quadrants individually as well as combined (p<0.05) |

(Continues)

| Authors and year | Sample (M/F) | Age group (years) | Population | Print collection method/Analysis method | Classification/  Lip area analyzed | Method of statistical analysis | Results | |
| --- | --- | --- | --- | --- | --- | --- | --- | --- |
|  |  |  |  |  |  |  | **Are there differences between sexes?** | **Description** |
| Kumar *et al.*, 2017 (10) | 119  (65/54) | 20-28 | North India | 1/Direct | S&T/Middle segment of the LL | Chi-square Test | 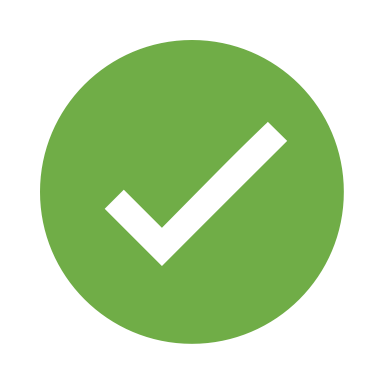 | 37% of males: type III;  52% of females: type I;  There are very highly sig. differences between sexes (p<0.001) |
| Dey *et al.*, 2019 (11) | 280 (138/142) | >15 | Oraon tribals and Bengalee Hindus communities, West Bengal, India | 1/Direct | S&T/4 quadrants | Chi-square Test | 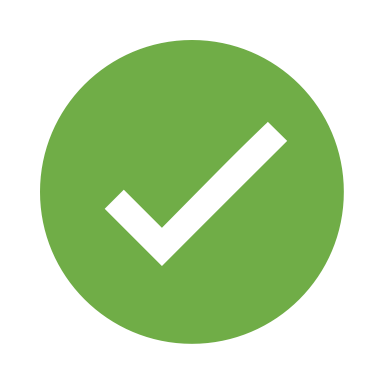 | Oraons: 60% of M and 62.9% of F with type II in Q1; 56% of M and 51.6% of F with type II in Q2; 52% of M with type II+III and 32.3% of F with type II or II+III in Q3; 60% of M with type II+III and 33.9% of females with type II in Q4; there are statistically sig. differences between sexes only in Q4 (ꭓ^2^=14.39; p<0.05);  Bengalee Hindus: 36.4% of M and 30% of F with type II in Q1; 28.4% of M and 27.5% of F with type II in Q2; 29.5% of M with type II+III and 38.8% of F with type III in Q3; 28.4% of M with type II+III and 35% of F with type III in Q4; there are statistically sig. differences between sexes in Q3 (ꭓ^2^=24.07; p<0.05) and 4 (ꭓ^2^=27.65; p<0.01);  There are variations in lip pattern between sexes in the study population |
| Gurung *et al.*, 2019 (12) | 205 (141/64) | 17-24 | Nepal | 2/Direct | S&T/4 quadrants | Descriptive analysis | 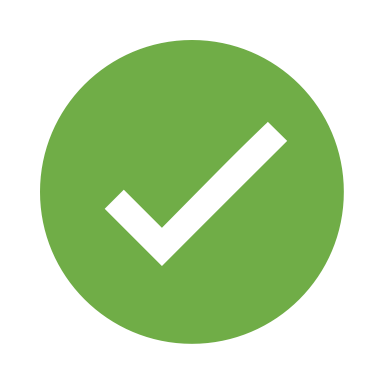 | Males: 32.6% with type I in RUQ; 27.7% with type I in LUQ; 34.8% with type II in LLQ and 31.2% with type II in RLQ;  Females: 29.7% with type I in RUQ; 29.7% with type II in LUQ; 48.4% with type I in LLQ and 42.2% with type I in RLQ;  There is variation in the frequency and distribution of lip patterns according to quadrant and sex |
| Anu *et al.*, 2020 (13) | 500 (250/250) | 18-40 | Chennai, India | 1/Direct | S&T/6 segments | Chi-square Test | 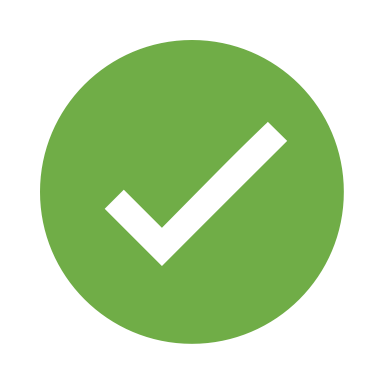 | There are statistically sig. differences between sexes (ꭓ^2^=37.65, p=0.05);  36.4% of males: type IV;  29.6% of females: type I; 29.6% of females: type II |

(Continues)

| Authors and year | Sample (M/F) | Age group (years) | Population | Print collection method/Analysis method | Classification/  Lip area analyzed | Method of statistical analysis | Results | |
| --- | --- | --- | --- | --- | --- | --- | --- | --- |
|  |  |  |  |  |  |  | **Are there differences between sexes?** | **Description** |
| Oliveira *et al.*, 2012 (14) | 104  (54/50) | - | Paraiba, Brazil | 4/Direct | S&T/8 segments | Chi-square Test; Fisher’s Exact Test | 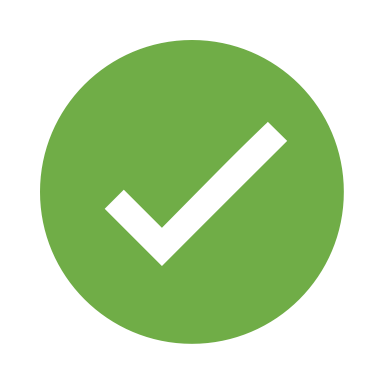 | 29.4% of the total male segments: type III;  28.5% of the total female segments: type II;  As for the type of groove, at the level of each segment, there are statistically significant differences between sexes only in segments 6 (p=0.016) and 8 (p=0.008); considering the whole lip, there are significant differences between sexes (p<0.001) |
| Sandhu *et al.*, 2012 (15) | 106  (56/50) | 18-25 | Punjab, India | 3/Direct | S&T/Middle segment of the LL | Z-Test | 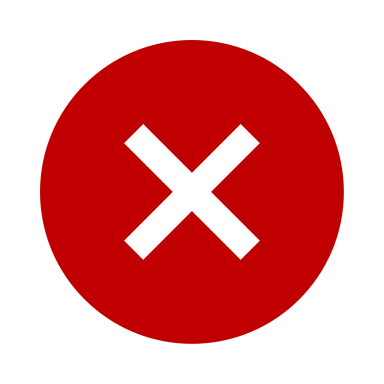 | 44.64% of males: type I;  40% of females: type I;  There are no statistically differences between sexes in individual lip print types (p>0.05) |
| Vats *et al.*, 2012 (16) | 1399 (781/618) | 8-60 | Brahmins, Jats and scheduled castes of Delhi and Haryana, India | 1/Direct | S&T modified/Whole | Z-Test | 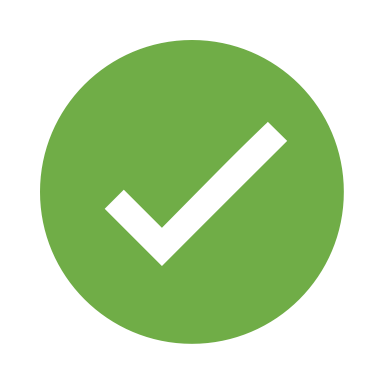 | Brahmins: 37.4% of males and 40% of females with type Y; there are sig. differences between sexes in patterns I’, II, III and IV (p<0.05);  Jats: 41.7% of males with type III and 33.2% of females with type Y; there are sig. differences between sexes in patterns I’, II, III, IV and Y (p<0.05);  Scheduled castes: 38.3% of males with type III and 30.8% of females with type Y; there are sig. differences between sexes in patterns I, I’, II, III and V (p<0.05) |
| Hammad *et al.*, 2014 (17) | 100  (27/73) | 19-25 | Lahore, Pakistan | -/- | S&T/Whole | Chi-square Test | 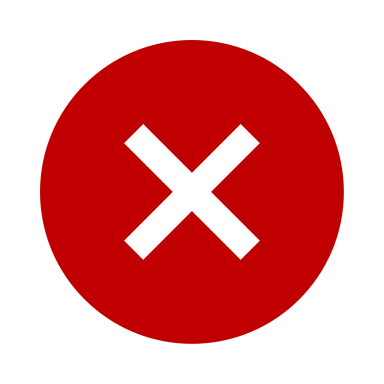 | 15 males and 45 females: type I;  No significant association was found between lip pattern and sex (ꭓ^2^=1.319; p=0.933) |

(Continues)

| Authors and year | Sample (M/F) | Age group (years) | Population | Print collection method/Analysis method | Classification/  Lip area analyzed | Method of statistical analysis | Results | |
| --- | --- | --- | --- | --- | --- | --- | --- | --- |
|  |  |  |  |  |  |  | **Are there differences between sexes?** | **Description** |
| Multani *et al.*, 2014 (18) | 200 (100/100) | 15-55 | Immigrant population of Rajnandgaon, India | 1/Direct | S&T and  Vahanwala *et al.*/Middle segment of the LL | Chi-square Test | 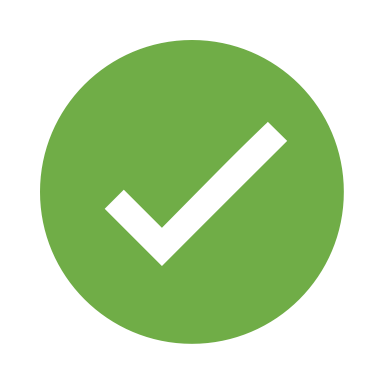 | Group 1 (15-25 years): 35.2% of males with type III; 58.6% of females with type I; 79.4% of males and 96.5% of females correctly identified;  Group 2 (26-35 years): 44.1% of males with type III; 51.1% of females with type I; 82.3% of males and 93% of females correctly identified;  Group 3 (36-45 years): 52.9% of males with type IV; 86.9% of females with type I; 94.1% of males and 86.9% of females correctly identified;  Group 4 (>45 years): 53.3% of males with type III; 40% of females with type I’; 80% of males and of females correctly identified;  Very highly sig. differences between sexes (p<0.0001) in the entire population (ꭓ^2^=13.53) and in groups 1 (ꭓ^2^=11.64) and 3 (ꭓ^2^=10.43);  Sig. differences between sexes (p<0.001) in groups 2 (ꭓ^2^=8.32) and 4 (ꭓ^2^=7.84);  Accuracy of cheiloscopy in sex estimation: 87.95%, 87.65%, 90.5% and 80% in group 1, 2, 3 and 4, respectively |
| Devi *et al.*, 2015 (19) | 225 (100/125) | - | Rajasthan, India | 4/Direct | S&T/12 segments | Independent T-test | 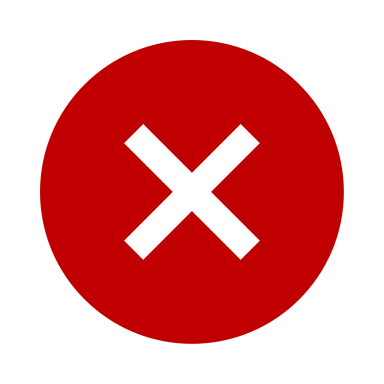 | 46% of the total male segments: type III;  48.6% of the total female segments: type III;  There are no sig. differences between sexes (p>0.05) |
| Peeran *et al.*, 2015 (20) | 104  (37/67) | 18-35 | Sebha, Libya | 2/Direct | S&T modified/4 quadrants | Chi-square Test | 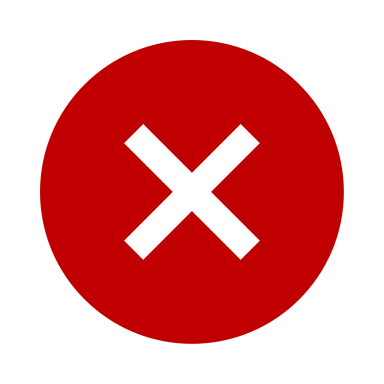 | 53.37% of the total male quadrants: type I;  60.07% of the total female quadrants: type I;  There are no statistically sig. differences between sexes in all quadrants: LU: (ꭓ^2^=5.357, p>0.05); RU: (ꭓ^2^=2.376, p>0.05); RL: (ꭓ^2^=3.23, p>0.05); LL: (ꭓ^2^=3.623, p>0.05) |

(Continues)

| Authors and year | Sample (M/F) | Age group (years) | Population | Print collection method/Analysis method | Classification/  Lip area analyzed | Method of statistical analysis | Results | |
| --- | --- | --- | --- | --- | --- | --- | --- | --- |
|  |  |  |  |  |  |  | **Are there differences between sexes?** | **Description** |
| Abdel Aziz *et al.*, 2016 (21) | 120  (60/60) | 22.37±1.79;  22.0±0.74;  22.30±0.84 | Egypt and Malaysia | 2/Direct | S&T/4 quadrants | Chi-square Test | 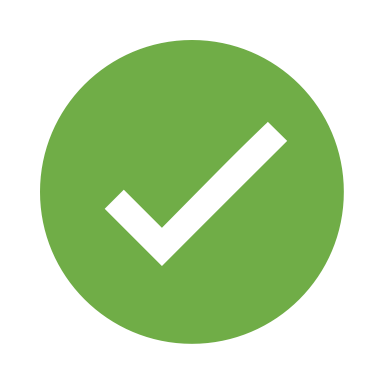 | Egypt population: 28.3% of the total male quadrants: type II; 28.3% of the total male quadrants: type III; 46.7% of the total female quadrants: type III; there are sig. differences between sexes (ꭓ^2^=13.347; p=0.020);  Malaysian population: 41.7% of the total male quadrants: type III; 30.8% of the total female quadrants: type II; there are no differences between sexes (ꭓ^2^=7.507; p=0.186);  At the quadrant level: there are sig. sex differences in Q3 (ꭓ^2^=12.616; p=0.008) and Q4 (ꭓ^2^=14.156; p=0.005) in the Egypt population; there are sig. sex differences in Q2 (ꭓ^2^=17.498; p=0.001) in the Malaysian population |
| Sehrawat, 2016 (22) | 106  (64/42) | 23.3 and 24.5 (mean) | Chandigarh, India | 1/Direct and indiret | S&T/4 quadrants | Chi-square Test | 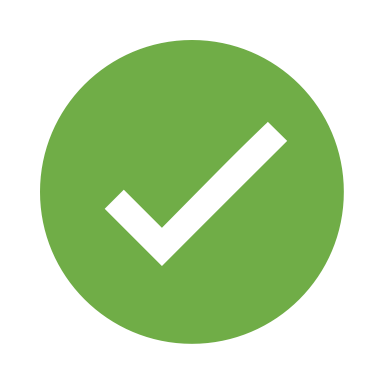 | 53.13% of males: type II;  35.72% of females: type II;  There are statistically sig. differences between sexes (ꭓ^2^=20.23; p=0.0004) |
| Bharat Kumar, 2017 (23)* | 200 (100/100) | 10-16 | Pathancheru, India | 3/Direct | S&T/4 quadrants | Descriptive analysis | 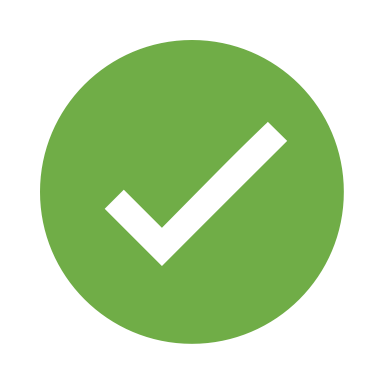 | 33.5% of male quadrants and 28% of female quadrants: intersected type;  Males: intersected type in Q1 (29%), Q3 (44%) and Q4 (34%);  Females: intersected type in Q1 (23%) and Q2 (45%) and branched type in Q3 (42%) and Q4 (34%) |
| Chaudhari *et al.*, 2017 (24) | 150  (75/75) | 25-50 | Chhattisgarh, India | 1/Direct | S&T and Vahanwala *et al.*/4 quadrants | Descriptive analysis | 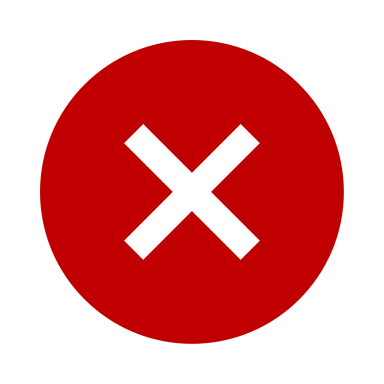 | 28.6% of the total male quadrants and 30.6% of the total female quadrants: type I;  42.6% of males with type I in Q3, 38.6% in Q4, 37.3% in Q1 and 30.6% in Q2;  41.3% of females with type I in Q2, 38.6% in Q4, 36% in Q3 and 32% in Q1;  No sex wise distribution was observed among lip print pattern |

(Continues)

| Authors and year | Sample (M/F) | Age group (years) | Population | Print collection method/Analysis method | Classification/  Lip area analyzed | Method of statistical analysis | Results | |
| --- | --- | --- | --- | --- | --- | --- | --- | --- |
|  |  |  |  |  |  |  | **Are there differences between sexes?** | **Description** |
| Manikya *et al.*, 2018 (25) | 180  (90/90) | 18-23 | Karnataka, Kerala and Manipur, India | 3/Direct | S&T and  Vahanwala *et al.*/6 segments | Chi-square Test | 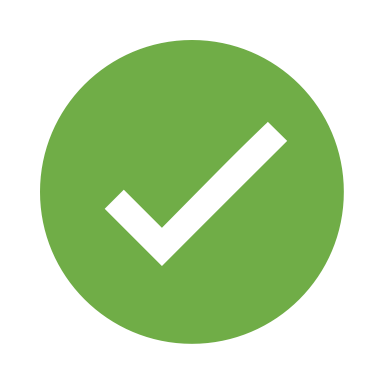 | 54.4% of males: type III;  30% of females: type I;  There are statistically significant differences between sexes in type I (p=0.003) and III (p=0.0003);  Kernataka: 53.3% of males with type III and 50% of females with type II; there are statistically sig. differences between sexes in type III (p=0.03);  Kerala: 63% of males with type III and 33% of females with type I; there are statistically sig. differences between sexes in type III (p=0.004);  Manipur: 47% of males with type III and 43% of females with type I; there are statistically sig. differences between sexes in type I (p=0.02);  61% of males and 59% of females correctly identified |
| Yendriwati *et al.*, 2019 (26) | 30  (15/15) | 20-26 | Malaysian with Chinese Ethnicity | 1/Direct | S&T/4 quadrants | Chi-square Test | 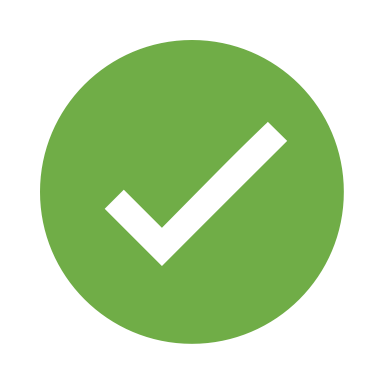 | 30% of males with type II in Q1, 43.3% in Q2, 23.3% in Q3 and 30% in Q4;  26.7% of females with type II in Q1, 30% in Q2, 26.7% in Q3 and 33.3% with type IV in Q4;  36.7% of males: type II;  30% of females: type IV  There are sig. differences in the predominant pattern between sexes (p=0.048); this difference is in type IV (p=0.007) |
| Maheswari and Gnanasundaram, 2011 (27) | 750 (348/402) | 3-70 | Chennai, India | 4/Direct | S&T modified/12 segments | Descriptive analysis | 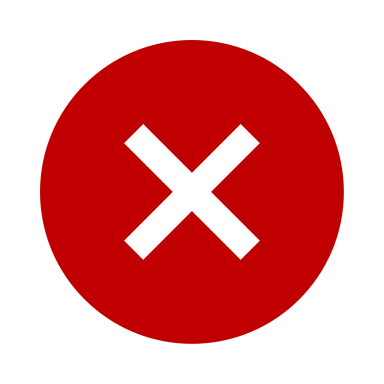 | 31.61% of males: type II;  43.79% of females: type II;  No peculiarity of lip print was established in males and females |

(Continues)

| Authors and year | Sample (M/F) | Age group (years) | Population | Print collection method/Analysis method | Classification/  Lip area analyzed | Method of statistical analysis | Results | | | |
| --- | --- | --- | --- | --- | --- | --- | --- | --- | --- | --- |
|  |  |  |  |  |  |  | **Are there differences between sexes?** | **Description** | |  |
| Karki, 2012 (28) | 150  (75/75) | 18-25 | Dhulikhel, Nepal | 4/- | S&T/4 quadrants | Descriptive analysis | 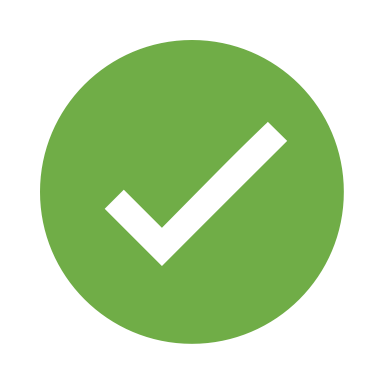 | | 38% of males: type I and I’;  42.5% of females: type II;  There are sig. differences between sexes | |
| Jatti and Rastogi, 2015 (29) | 150  (75/75) | 18-70 | Coimbatore, India | 1/Indirect | S&T/4 quadrants | Descriptive analysis | 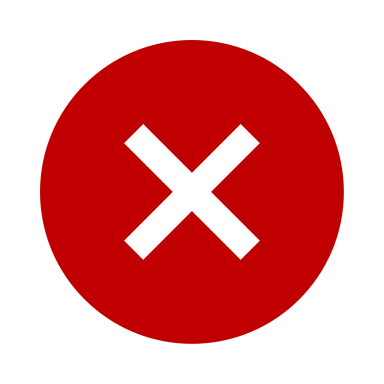 | | Type II: most common pattern in the UL and LL of both sexes;  Males: type II is most common in all quadrants with 42%, 55%, 45% and 48% in quadrants 1 to 4, respectively;  Females: type II is most common in all quadrants with 49%, 42%, 45% and 47% in quadrants 1 to 4, respectively;  No sig. differences were found in lip patterns between sexes | |
| Verma *et al.*, 2015 (30) | 100  (50/50) | 15-35 | *-* | 1/Direct | S&T modified/Middle segment of the LL | Chi-square Test | 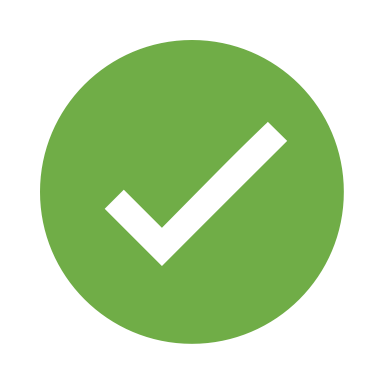 | | 36% of males: vertical pattern; 36% of males: reticular pattern;  44% of females: branched pattern;  Statistically significant association between lip pattern and sex (ꭓ^2^=16.68; p=0.002) | |
| Šimović *et al.*, 2016 (31) | 90  (40/50) | - | Croatia | 4/Direct | S&T modified/Whole | Chi-square Test; Fisher’s Exact Test | 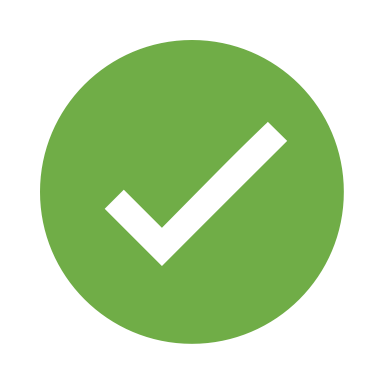 | | 35% of males: type 3 (dotted lines); 40% of females: type 2 (branched lines);  The Chi-square Test showed a statistically sig.  difference between sexes (p<0.05); the variables of sex and  groove types are dependent;  The Fisher’s Exact Test also showed a statistically significant difference between sexes (p<0.05; Z=1.96) | |

(Continues)

| Authors and year | Sample (M/F) | Age group (years) | Population | Print collection method/Analysis method | Classification/  Lip area analyzed | Method of statistical analysis | Results | |
| --- | --- | --- | --- | --- | --- | --- | --- | --- |
|  |  |  |  |  |  |  | **Are there differences between sexes?** | **Description** |
| Dixit *et al.*, 2019 (32) | 20  (10/10) | - | Indian and african | 4/Direct | S&T/4 quadrants | Descriptive analysis | 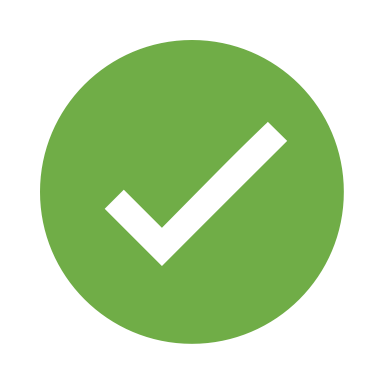 | Caucasoid: 40% of M with type I and 40% of F with type I or I’ in LU*;* 40% of M with type I and 60% of F with type I’ in LL; 40% of M with type I or I’ and 40% of F with type II in RU; 40% of M with type II and 60% of F with type I in RL;  Negroid: 80% of M with type I’ and 60% of F with type I in LU; 60% of M with type I and 60% of F with type II in LL; 40% of M with type I’ or II and 60% of F with type I’ in RU; 60% of M with type I’ and 60% of F with type II in RL;  Depending on the frequency of patterns occurring in each quadrant, one can also relate it to sexual dimorphism |
| Priya *et al.*, 2019 (33) | 102  (52/50) | 18-30 | 18 different nationalities^(1)^ | 1/- | S&T/8 segments | Chi-square Test | 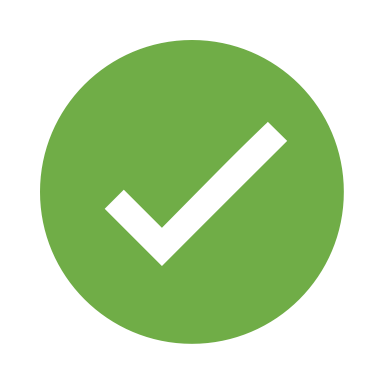 | There are no significant differences between sexes; there are only in RU1 (right segment of the middle part of the UL) (p=0.001) and LL1 (left segment of the middle part of the LL) (p=0.04) |
| Vaishnavi *et al.*, 2019 (34) | 50  (25/25) | 15-20 | Chennai, India | 1/Direct | Classification developed by the authors^(2)^/4 quadrants | Descriptive analysis | 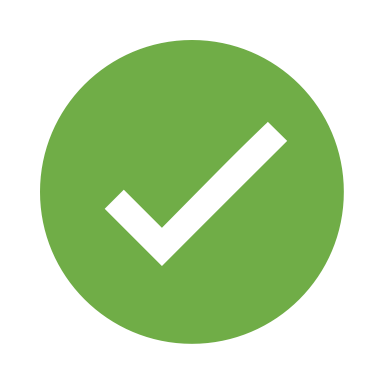 | There is variation in lip pattern between sexes |
| Yandava *et al.*, 2020 (35)* | 196 (78/118) | 17-20 | Hyderabad, India | 2/Direct | S&T/4 quadrants | Descriptive analysis | 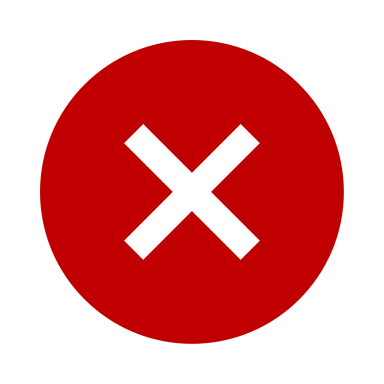 | 91%, 91%, 92% and 87% of males with type I’ in quadrants 1, 2, 3 and 4, respectively;  87%, 83%, 84% e 83% of females with type I’ in quadrants 1, 2, 3 and 4, respectively |
| Negi and Negi, 2016 (36) | 200 (100/100) | - | North India | 3/Direct | Nagasupriya *et al.*^(3)^/Middle segment of the LL | Chi-square Test | 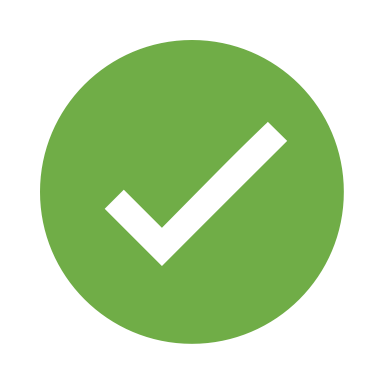 | 56% of males: type II;  38% of females: type I;  The distribution of lip patterns was statistically significant in males (ꭓ^2^= 13.480; p=0.001) and statistically insignificant in females (ꭓ^2^=0.520; p=0.771) |

(Continues)

| Authors and year | Sample (M/F) | Age group (years) | Population | Print collection method/Analysis method | Classification/  Lip area analyzed | Method of statistical analysis | Results | |
| --- | --- | --- | --- | --- | --- | --- | --- | --- |
|  |  |  |  |  |  |  | **Are there differences between sexes?** | **Description** |
| Ahuja *et al.*, 2018 (37)* | 100  (50/50) | 18-25 | Gujarat, India | 1/Direct | S&T/4 quadrants | Descriptive analysis | 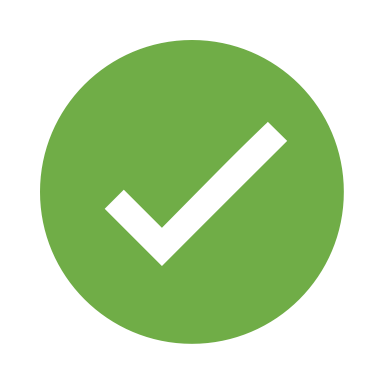 | 35.60% of the total male quadrants: type II;  28.28% of the total female quadrants: type I |
| Bai *et al.*, 2018 (38) | 300 (150/150) | 18-25 | - | 3/Direct | S&T/Middle segment of the LL | Chi-square Test | 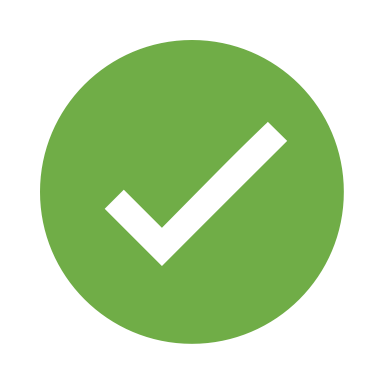 | 59 males with type II;  50 females with type I;  There are very high statistical significance for different lip patterns between males and females (ꭓ^2^=23.98; p<0.0001) |
| Padmavathi *et al.*, 2013 (39) | 250  (-/-) | - | Gujarat and Rajasthan, India | 1/Indirect | Classification developed by the authors^(4)^/ Middle segment of the UL and LL | Chi-square Test | 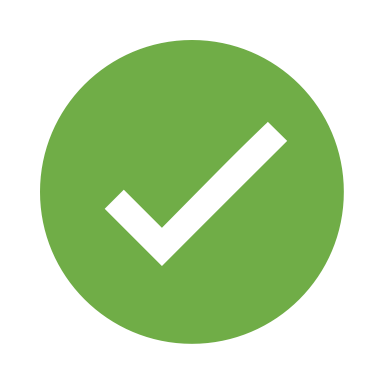 | There are no sig. differences between sexes in patterns CV, IV and B (p>0.05);  There are sig. differences between sexes in patterns D, R and CP, only on the UL (p<0.05);  Only the upper lip pattern is a good sex discriminator |
| Ishaq *et al.*, 2018 (40) | 250  (125/125) | - | Lahore, Pakistan | 1/- | S&T/Whole | Descriptive analysis | 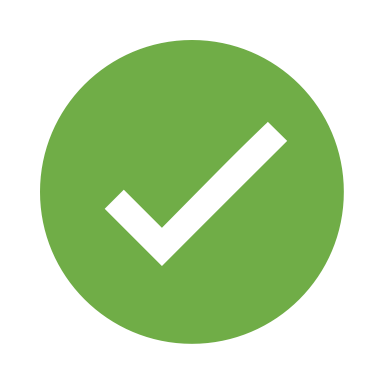 | 76 males: type III  84 females: type I;  Lip print patterns show variation according to the sex |
| Sagar *et al.*, 2019 (41)* | 200 (100/100) | Different age groups | Different  ethnic backgrounds, Eastern India | -/- | S&T/Middle segment of the LL | Descriptive analysis | 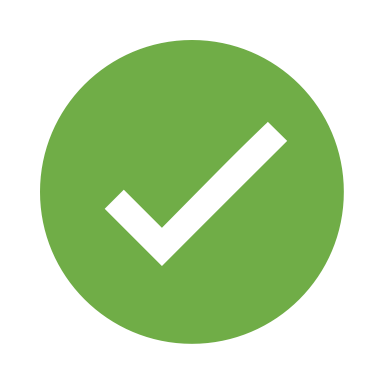 | 28% of males: type I’;  35% of females: type I |

^(1)^ Bahrain, Egypt, Iran, Iraq, Jordon, Kuwait, Oman, Palestine, Saudi, Sudan, Syria, United Arab Emirates, India, Pakistan, America, Canada, New Zealand and Tanzania.

^(2)^ Type 1 – complete vertical grooves; Type 2 – partial vertical grooves; Type 3 – branched grooves; Type 4 – undetermined patterns.

^(3)^ Type I – Vertical pattern (grooves running vertically to full length or partially across the lips); Type II – Branched pattern (grooves exhibiting branching); Type III – Reticular pattern (Grooves intersecting or crisscrossing each other).

^(4)^ CV (Complete Vertical); IV (Incomplete Vertical); B (Branched); R (Reticular); D (Dots); CP (Complex Pattern).

M/F – Male/Female; UL – Upper Lip; LL – Lower Lip; sig. – significant; Q – Quadrant; LU –Left Upper; RU – Right Upper; LL – Left Lower; RL – Right Lower.

“-“ – The variable was not reported by the study authors.

The existence or inexistence of differences between sexes in the articles marked with an asterisk (*) was assumed, considering the results described by the respective authors.

- Yes.


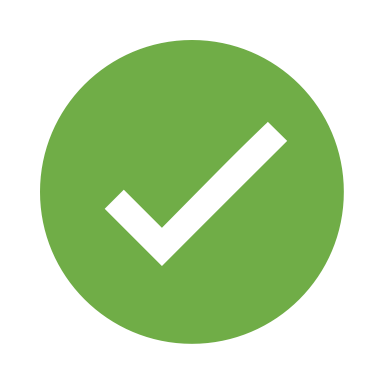

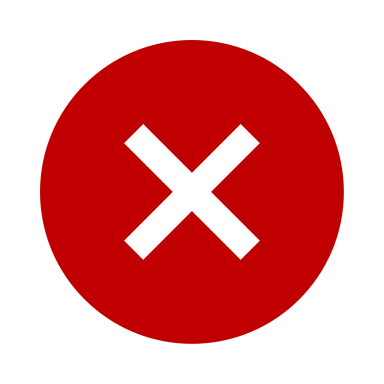


- No.

- Sim.


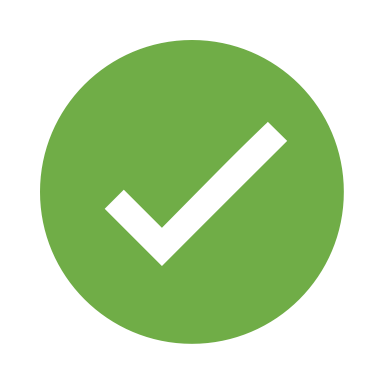

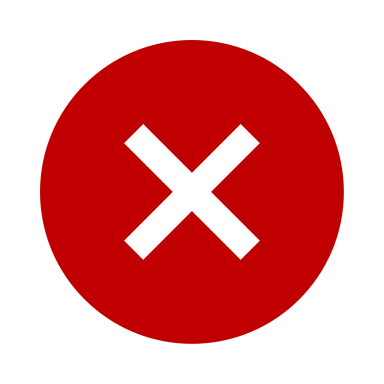


- Não.
