# Supplementary material for: Cheiloscopy in sex estimation: a systematic review
Source: Forensic Sci Med Pathol. 2023 May 27;20(1):280–92. doi: 10.1007/s12024-023-00648-9 (PMC10944408; doi:10.1007/s12024-023-00648-9)
Supplement: Supplementary file 2 — Supplementary file1 (DOCX 62 KB) [file 12024_2023_648_MOESM2_ESM.docx]

Supplementary table 2 - Risk of bias for each included study.

| Authors and year | Definition of the aim | Description of the population data | Presentation of the inclusion or exclusion criteria of the participants | Presentation of the methodology | Assessment of reliability | | Statistical analysis | Presentation of results | Answer to the aim | Rationale of the conclusions |
| --- | --- | --- | --- | --- | --- | --- | --- | --- | --- | --- |
|  |  |  |  |  | **Intra-rater** | **Inter-rater** |  |  |  |  |
| Maheswari and Gnanasundaram, 2011 [47] | ↓ | ↓ | ↓ | → | ● | ● | → | → | ↓ | ↓ |
| Randhawa *et al.*, 2011 [24] | ↓ | ↓ | ↓ | ↓ | ↓ | ↓ | → | → | ↓ | ↓ |
| Karki, 2012 [48] | ↓ | ↓ | ↓ | → | ● | ● | → | → | ↓ | ↓ |
| Oliveira *et al.*, 2012 [34] | ↓ | → | ↓ | ↓ | ● | ↓ | → | → | ↓ | ↓ |
| Sandhu *et al.*, 2012 [35] | ↓ | ↓ | ↓ | ↓ | ● | ● | → | → | ↓ | ↓ |
| Vats *et al.*, 2012 [36] | ↓ | ↓ | ↓ | → | ● | ● | → | ↓ | ↓ | ↓ |
| Padmavathi *et al.*, 2013 [57] | ↓ | ↑ | ↓ | ↑ | ● | ● | → | → | ↑ | ↓ |
| Hammad *et al.*, 2014 [37] | ↓ | ↓ | ↓ | → | ● | ● | → | ↓ | ↓ | ↓ |
| Multani *et al.*, 2014 [38] | ↓ | ↓ | ↓ | ↓ | ● | → | → | → | ↓ | ↓ |
| Devi *et al.*, 2015 [39] | ↓ | → | ↓ | ↓ | ● | **NA** | → | → | ↓ | ↓ |
| Jatti e Rastogi, 2015 [49] | ↓ | ↓ | ↓ | ↓ | ● | ● | → | → | → | ↓ |
| Mantilla Hernández *et al.*, 2015 [25] | ↓ | ↓ | ↓ | ↓ | ● | **NA** | → | ↓ | ↓ | ↓ |
| Peeran *et al.*, 2015 [40] | ↓ | ↓ | ↓ | → | ● | ● | → | ↓ | ↓ | ↓ |
| Ramakrishnan *et al.*, 2015 [17] | ↓ | ↓ | ↓ | ↓ | ● | ↓ | → | ↓ | ↓ | ↓ |
| Verma *et al.*, 2015 [50] | ↓ | → | ↓ | → | ● | ↑ | → | ↓ | ↓ | ↓ |

(Continues)

| Authors and year | Definition of the aim | Description of the population data | Presentation of the inclusion or exclusion criteria of the participants | Presentation of the methodology | Assessment of reliability | | Statistical analysis | Presentation of results | Answer to the aim | Rationale of the conclusions |
| --- | --- | --- | --- | --- | --- | --- | --- | --- | --- | --- |
|  |  |  |  |  | **Intra-rater** | **Inter-rater** |  |  |  |  |
| Abdel Aziz *et al.*, 2016 [41] | ↓ | ↓ | ↓ | ↓ | ● | ● | → | → | ↓ | ↓ |
| Badiye and Kapoor, 2016 [26] | ↓ | ↓ | ↓ | ↓ | → | → | → | ↓ | ↓ | ↓ |
| Moshfeghi *et al.*, 2016 [27] | ↓ | ↓ | ↓ | → | ↓ | ↓ | → | → | ↓ | ↓ |
| Negi and Negi, 2016 [54] | ↓ | → | ↑ | ↑ | ● | ● | → | ↓ | ↓ | ↓ |
| Sehrawat, 2016 [42] | ↓ | ↓ | ↓ | ↓ | ● | ● | → | → | ↓ | ↓ |
| Šimović *et al.*, 2016 [51] | ↓ | → | ↓ | → | ● | ● | → | ↓ | ↓ | ↓ |
| Tarvadi and Goyal, 2016 [28] | ↓ | ↓ | ↓ | ↓ | ● | ● | → | ↓ | ↓ | ↓ |
| Basheer *et al.*, 2017 [29] | ↓ | ↓ | ↓ | ↓ | ● | ● | → | ↓ | ↓ | ↓ |
| Bharat Kumar, 2017 [43] | ↓ | ↓ | ↓ | ↓ | → | ● | → | → | ↓ | ↓ |
| Chaudhari *et al.*, 2017 [44] | ↓ | ↓ | ↓ | ↓ | ● | ● | → | → | ↓ | ↓ |
| Kapoor and Badiye, 2017 [18] | ↓ | ↓ | ↓ | ↓ | → | → | → | ↓ | ↓ | ↓ |
| Kumar *et al.*, 2017 [30] | ↓ | ↓ | ↓ | ↓ | ● | **NA** | → | → | ↓ | ↓ |
| Ahuja *et al.*, 2018 [55] | ↓ | ↓ | ↑ | ↓ | ● | ● | → | → | → | ↓ |
| Bai *et al.*, 2018 [56] | ↓ | → | ↓ | → | ● | ● | → | → | ↓ | ↓ |
| Herrera *et al.*, 2018 [8] | ↓ | ↓ | ↓ | ↓ | ● | ● | ↓ | ↓ | ↓ | ↓ |

(Continues)

| Authors and year | Definition of the aim | Description of the population data | Presentation of the inclusion or exclusion criteria of the participants | Presentation of the methodology | Assessment of reliability | | Statistical analysis | Presentation of results | Answer to the aim | Rationale of the conclusions |
| --- | --- | --- | --- | --- | --- | --- | --- | --- | --- | --- |
|  |  |  |  |  | **Intra-rater** | **Inter-rater** |  |  |  |  |
| Ishaq *et al.*, 2018 [58] | ↑ | → | ↓ | → | ● | ● | → | → | ↓ | ↓ |
| Manikya *et al.*, 2018 [45] | ↓ | ↓ | ↓ | ↓ | ● | ● | → | → | ↓ | ↓ |
| Dey *et al.*, 2019 [31] | ↓ | ↓ | ↓ | ↓ | ● | ● | → | ↓ | ↓ | ↓ |
| Dixit *et al.*, 2019 [12] | ↓ | → | ↑ | ↓ | ● | ● | → | ↓ | ↓ | ↓ |
| Gurung *et al.*, 2019 [32] | ↓ | ↓ | ↓ | ↓ | ● | ● | → | ↓ | ↓ | ↓ |
| Priya *et al.*, 2019 [52] | ↓ | ↓ | ↓ | → | ● | ● | → | → | ↓ | ↓ |
| Sagar *et al.*, 2019 [59] | → | → | ↓ | ↑ | ● | ● | → | ↓ | ↑ | ↓ |
| Vaishnavi *et al.*, 2019 [53] | ↓ | ↓ | ↓ | ↑ | ● | ● | → | → | ↓ | ↓ |
| Yendriwati *et al.*, 2019 [46] | ↓ | ↓ | ↓ | ↓ | ● | ● | → | → | ↓ | ↓ |
| Anu *et al.*, 2020 [33] | ↓ | ↓ | ↓ | ↓ | ● | ● | → | ↓ | ↓ | ↓ |
| Yandava *et al.*, 2020 [7] | ↓ | ↓ | ↓ | ↓ | ● | ● | → | ↓ | ↑ | → |

**NA -**  Not Applicable ● - Uncertain risk of bias ↑ - High risk of bias → - Medium risk of bias ↓ - Low/null risk of bias
